# Resolution of epoetin‐induced pure red cell aplasia, successful re‐challenge with roxadustat

**DOI:** 10.1111/ijlh.13325

**Published:** 2020-08-27

**Authors:** Yunzhou Wu, Xudong Cai, Jianing Ni, Xiaomeng Lin

**Affiliations:** ^1^ Department of Nephrology Ningbo Chinese Medical Hospital Affiliated to Zhejiang Chinese Medical University Ningbo China

**Keywords:** anti‐erythropoietin antibody, haemoglobin, pure red cell aplasia, roxadustat, unusual case

## Abstract

The application of erythropoietin (EPO) can bring about a rare but serious complication called anti‐EPO antibody‐mediated pure red cell aplasia (PRCA). Once the disease is diagnosed, EPO administration should be stopped immediately. However, after the removal of the anti‐EPO antibody, treating anaemia in these patients with chronic renal disease with EPO therapy is difficult, as restarting EPO therapy risks the recurrence of anti‐EPO antibody‐mediated PRCA. A 26‐year‐old man with anaemia related to renal failure, who was administered recombinant human EPO subcutaneously, developed anti‐EPO antibody‐mediated PRCA. After removal of antibodies by treatment with corticosteroids and cyclosporine, therapy for anaemia of chronic renal disease with roxadustat achieved good results. Roxadustat is a new type of drug for the treatment of anaemia, and it can stimulate endogenous EPO within or near the physiologic range and increase haemoglobin levels.

## 1 INTRODUCTION

As recombinant human erythropoietin (rHuEPO) was introduced in the 1980s, it has proved as a breakthrough in the treatment of anaemia of chronic renal disease and has freed patients on maintenance dialysis from long‐term dependence on blood transfusion. However, rHuEPO can cause pure red cell aplasia (PRCA) in any age group, particularly in patients receiving specific rHuEPO with subcutaneous administration of epoetin‐α (Eprex or Erypo [Janssen; Beerse, Belgium]) marketed outside the USA, particularly in Europe.^1^ PRCA is a rare type of severe anaemia characterized by a very low reticulocyte count and almost no erythroid precursor cells in the bone marrow, and treatment is difficult for this condition.

rHuEPO is generally discontinued after a diagnosis of PRCA. While a few patients can experience spontaneous remission, most require immunosuppressive therapy. Whether rHuEPO can be resumed after immunosuppressive therapy is controversial. rHuEPO treatment has successfully improved haemoglobin levels once anti‐EPO antibodies were removed, but there is a risk of recurrence.^2^ This case report shows the successful treatment of a patient with anti‐EPO antibody‐mediated PRCA with roxadustat, an oral hypoxia‐inducible factor prolyl hydroxylase inhibitor, to increase haemoglobin after the removal of immunosuppressive antibodies.

## 2 CASE REPORT

A 26‐year‐old man with uraemia was diagnosed with anti‐EPO antibody‐mediated PRCA after receiving subcutaneous rHuEPO for 12 months. In 2018, this patient was hospitalized for uraemia treated with haemodialysis initially and peritoneal dialysis subsequently. The possible reason for uraemia was chronic glomerulonephropathy, but a renal biopsy was not performed. Upon initiation of dialysis, his haemoglobin was 5.2 g/dL. rHuEPO (epoetin‐α, 10 000 international units [IU]; Epogen^®^, 3SBio; Shenyang, China) was administered subcutaneously once a week with an increase in haemoglobin to approximately 11 g/dL over a period of 10 months. In January 2019, the patient's haemoglobin suddenly dropped to 6.7 g/dL. Despite an increase in the rHuEPO dosage to 10 000 IU subcutaneously twice per week, his haemoglobin declined to 5.4 g/dL along with symptoms such as significant fatigue and chest tightness, requiring blood transfusions to maintain a haemoglobin level of approximately 6.0 g/dL. When admitted to our hospital in February 2019, no obvious abnormalities in a right ilium marrow biopsy were found. rHuEPO treatment and a blood transfusion of 200 mL once per week were continued without change in haemoglobin. To clarify the cause of anaemia, the patient was re‐admitted to our hospital in April 2019.

The blood test results were as follows: red blood cells 149 × 10^4^/μL, haemoglobin 4.4 g/dL, haematocrit 12.7%, reticulum percentage 0.1%, platelet count 9.7 × 10^4^/μL. The results of the second bone marrow biopsy showed proliferative active bone marrow with a rare red line. The diagnosis of PRCA was confirmed.

The diagnoses of thymoma, systemic autoimmune disease and parvovirus B19 infection were ruled out. The patient was not treated with drugs such as phenytoin or chloramphenicol. Based on the findings mentioned above, we strongly suggested that the patient's PRCA was caused using rHuEPO. Therefore, we conducted an anti‐EPO antibody test on the patient, which was not routinely performed in our hospital. Anti‐EPO antibody testing by an enzyme‐linked immunosorbent assay method was performed (3SBio) to include an examination of neutralization activity in vitro, revealing high titres of anti‐EPO antibody (1:125) supporting the diagnosis of rHuEPO‐mediated PRCA.

**Figure 1:**
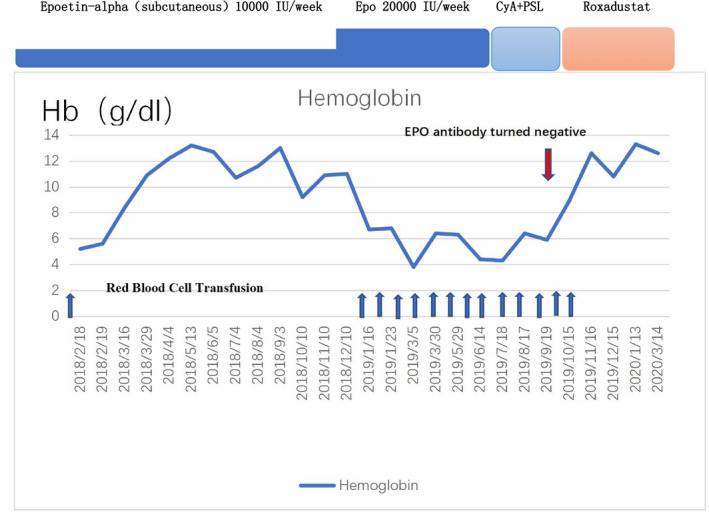
Clinical course in a patient with pure red cell aplasia (PRCA) induced by anti‐erythropoietin antibodies (anti‐EPO Ab). His haemoglobin (Hb) levels suddenly decreased in January 2019. There was no obvious abnormality in the first right ilium marrow biopsy, so EPO treatment was continued with increased dosage to 20 000 IU/wk. EPO therapy was ceased, and immunosuppressive therapy was initiated (cyclosporine + prednisolone for 3 mo) in June 2019 after diagnosis of epoetin‐induced pure red cell aplasia. Anti‐EPO antibody turned negative in September 2019. Roxadustat was applied to treat anaemia of chronic renal disease in October 2019. CyA, cyclosporine; PSL, prednisolone

After diagnosis, rHuEPO was discontinued, and 0.5 mg/kg/d prednisone (PSL) and 2 mg/kg/d cyclosporine (CyA) (divided in two doses) were prescribed. Weekly transfusion of 200 mL of blood was continued. PSL was tapered by 5 mg per week after 6 weeks owing to irritability and was discontinued. CyA was gradually increased to 3 mg/kg/d. The plasma concentration of CyA yielded a concentration ranging from 100‐200 ng/mL. Immunosuppressants were discontinued once anti‐EPO antibodies could no longer be detected. However, although anti‐EPO antibody tests were negative, the haemoglobin level still could not be maintained. It has been suggested that EPO can be reapplied after the patient's antibodies clear or fall below the critical value, with intravenous injection used as much as possible.^2^ However, we did not want to risk re‐injecting rHuEPO, which could lead to a recurrence of PRCA. EPO treatment has been effective in anti‐EPO antibody‐mediated PRCA patients whose anti‐EPO antibodies were negative after immunosuppressive therapy. We suggested that roxadustat might also be effective in these post‐treatment antibody‐negative patients. Therefore, 120 mg of roxadustat was administered orally three times a week. His haemoglobin started to stabilize and rose rapidly (Figure 1). Anti‐EPO antibody results remained negative 2 months after continued use of oral roxadustat.

## 3 DISCUSSION

This is the first case report of a hypoxia‐inducible factor prolyl hydroxylase inhibitor (HIF‐PHI) as an effective treatment for anaemia of chronic renal disease in PRCA patients in whom anti‐EPO antibody tests turn negative after immunosuppressive therapy.

PRCA should be suggested in patients with severe anaemia who previously responded to rHuEPO. The main features of anti‐EPO antibody‐mediated PRCA include rHuEPO treatment history, anti‐EPO antibody, severe anaemia, the virtual absence of reticulocytes, and limited red blood cell precursors in otherwise normal bone marrow.^3^ Stopping rHuEPO and initiating immunosuppressive therapy are the main treatment options. After anti‐EPO antibody is no longer detectable, treatment options are considered to avoid PRCA relapse. There was a case report of no recurrence, which was treated with an intravenous continuous EPO receptor stimulator (CERA) after EPO‐induced PRCA.^4^ However, CERA is not available in China. Peginesatide, a small‐molecule synthetic peptide, has also been reported to correct anaemia in patients with PRCA caused by anti‐EPO antibodies.^5^ Unfortunately, peginesatide was withdrawn from the market because of unexplained and serious adverse drug reactions.^6^

Roxadustat could also be a valid option. Roxadustat is a new type of drug for the treatment of anaemia, which is available in China and Japan. It is known that the kidneys of patients with kidney disease are still able to produce EPO. Roxadustat is an oral HIF‐PHI, which can stimulate endogenous EPO within or near the physiologic range and increases haemoglobin levels.^7^ Studies in China and Japan have shown it to be effective and safe．However, there were strict exclusion and inclusion criteria in these studies, and no patients with PRCA were included. In this patient, treatment with roxadustat after anti‐EPO antibody removal was successful. Anti‐EPO antibodies were still negative after oral roxadustat, and the patient's haemoglobin level was maintained. Roxadustat may be less immunogenic than rHuEPO for the following reasons.

There is a highly developed immune system in the skin, which has many different cell types contributing to immune responses and antigen presentation.^8^ The prolonged exposure of these cells to rHuEPO after subcutaneous administration may facilitate the generation of anti‐EPO antibodies. Roxadustat is an oral agent that avoids stimulating the subcutaneous immune system. Additionally, anti‐EPO antibodies have been demonstrated to be directed against specific epitopes of the protein moiety.^9^ Increased immunogenicity due to secondary changes induced by formulation or storage conditions can lead to the exposure of previously hidden epitopes or generation of immunogenic structures. Anti‐EPO antibodies developed in this way are cross‐reactive with endogenous EPO, which leads to more severe anaemia than that in the absence of rHuEPO therapy. Finally, roxadustat is a chemical drug, which has an entirely different chemical structure from rHuEPO. Roxadustat stimulates endogenous EPO at much lower peaks than in those treated with traditional rHuEPO.^10^

However, anti‐EPO antibodies may influence both endogenous and exogenous EPO, so roxadustat may not be effective in anti‐EPO antibody‐mediated PRCA patients who still have a high antibody titre. For patients with anti‐EPO antibody‐mediated PRCA who test negative for antibodies after immunosuppressive therapy, treatment with roxadustat may not only improve the patient's haemoglobin level, but also reduce the probability of PRCA recurrence.

In conclusion, it is important to pay attention to patients with anaemia of chronic renal disease who cannot maintain haemoglobin with subcutaneous injections of high‐dose rHuEPO. Additionally, it is also important to consider the possibility of anti‐EPO antibody‐mediated PRCA. We report for the first time that roxadustat can be effective in restarting therapy after antibody clearance. However, extensive research is needed to confirm these results.

## CONFLICT OF INTEREST

The authors thank 3SBio for measuring anti‐EPO antibody concentrations. The authors have no competing interests.
